# Effect of Cognitive Function on Depression Symptoms in Korean Older Adults Living Alone: Moderating Role of Gender

**DOI:** 10.1093/geroni/igaf122.098

**Published:** 2025-12-31

**Authors:** Si Young Song

**Affiliations:** Kyung Hee University, Syracuse, New York, United States

## Abstract

This study examines the effect of cognitive function on depression symptoms in older adults living alone and investigates the moderating role of gender. Data were obtained from the 2023 National Survey of Older Koreans, a nationally representative survey conducted in South Korea, focusing on individuals aged 65 and older who live alone. Cognitive function was assessed using the K-MMSE-2 (Korean Mini-Mental State Examination-2) scale (range: 0–30), and depression symptoms were measured with the SGDS-15 (Short Geriatric Depression Scale-15) (range: 0–13). Gender was analyzed as a moderating variable, while age, education level, household income, life satisfaction, and social contact frequency were controlled. A moderation analysis was conducted. The findings indicate that cognitive function is negatively associated with depression symptoms in older adults living alone. Moreover, gender significantly moderates this relationship. The simple slope test revealed that higher cognitive function is linked to lower depression symptoms in both male and female older adults living alone. However, the association was stronger in males, indicating that cognitive function plays a more critical role in mitigating depression symptoms among older men living alone. These results highlight the need for cognitive health interventions to reduce depression symptoms in older adults living alone. Mental health policies should incorporate gender-specific strategies, and community-based cognitive training programs should be expanded to promote emotional well-being in this population.

